# Lower left atrial strain in the presence of regional atrial fibrosis: an MRI study of patients with atrial fibrillation

**DOI:** 10.1186/1532-429X-18-S1-P207

**Published:** 2016-01-27

**Authors:** Dana C Peters, James S Duncan, Karl Grunseich, Mark Marieb, Daniel Cornfeld, Albert J Sinusas, Sudhakar Chelikani

**Affiliations:** 1 grid.47100.320000000419368710Diagnostic Radiology, Yale School of Medicine, New Haven, CT USA; 2 grid.47100.320000000419368710Cardiology, Yale School of Medicine, New Haven, CT USA

## Background

LA strain, measured by feature-tracking using cine MRI, is an emerging tool for regional evaluation of the left atrium [1-4]. Late gadolinium enhancement (LGE MRI) is a method which indicates regions of possible atrial fibrosis. Their regional relationship is not known. Our goal was to compare global and regional left atrial (LA) strain in atrial fibrillation (AF) subjects and controls, and relate strain globally to LA volumes and ejection fraction (EF), and regionally to the presence of fibrosis. Does LA strain reflect fibrosis?

## Methods

Eighteen patients with atrial fibrillation (AF) and 12 controls were imaged using 3D LGE and 2D cine MRI with the 2-chamber and 4-chamber views, on a Siemens 1.5 T Aera. The 3D LGE sequence was an inversion recovery spoiled gradient echo sequence with fat-saturation, ECG-gating, and right hemi-diaphragm navigator-gating. Spatial resolution was 1.4 × 1.4 × 3.2 mm^3^ resolution before zero-filling, TR/TE/θ=5.2ms/1.6ms/15°, 360 Hz/pixel, GRAPPA factor of 2. Atrial LGE volumes were measured, normalized to atrial myocardial volumes (LGE burden), using a CNR threshold of 3.5, and excluding artifacts. LA volumes and total LA ejection fraction were measured using the bi-plane method. Circumferential strain was estimated between atrial end-diastole and atrial end-systole, using a customized point-matching method for the left atrial contours at the two cardiac phases (maximum and minimum atrial areas). The contours were discretized into points, which were matched using an iterative process that relies on closest point matching, and then a piece-wise affine transformation. Circumferential strain was calculated, using the initial and final distances between neighboring matched points. Regional strain maps were generated, and compared to enhancement patterns on reformatted 4-chamber LGE. Strains in LGE enhanced regions were quantitatively compared to strains in unenhanced areas.

## Results

**Figure 1:**
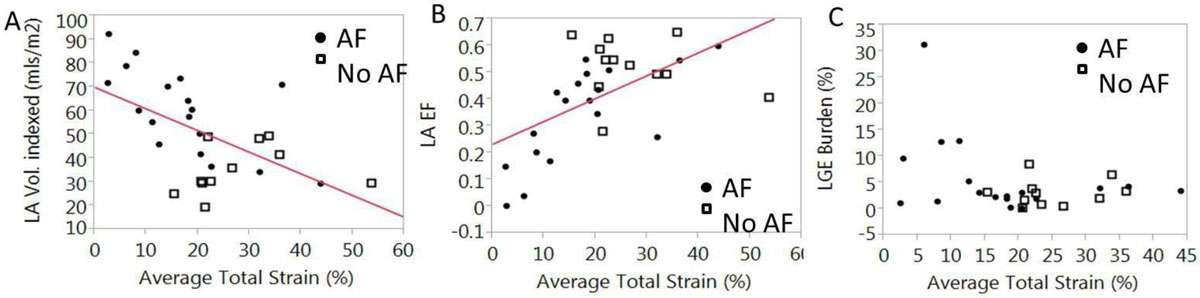
**A) Strain strongle correlates with LA EF (R=0.57, p < 0.001) and B) maximal LA volumes (R=-0.57, p = 0.001), but modestly with LGE burden (C) (R=-0.36, p = 0.049)**.

**Figure 2:**
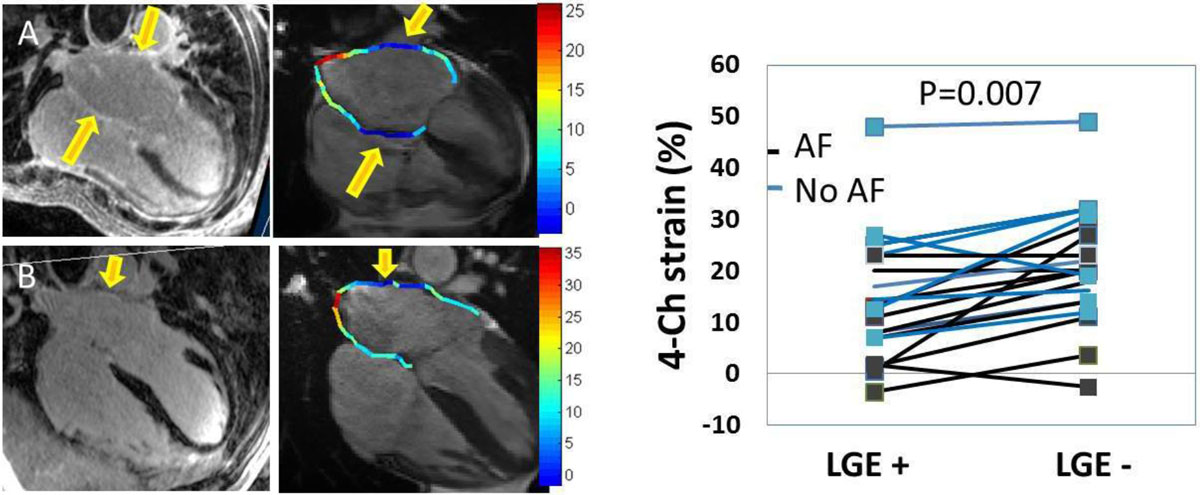
**Reformatted 3D LGE volumes show regional enhancement which corresponds to regional low strain**. A-B) Arrows show corresponding regions of LGE and low-strain in two AF subjects. C). The strain in LGE in enhanced regions was lower than in non-enhanced areas (14 ± 11% in LGE enhanced vs. 20 ± 12% in non LGE enhanced regions, p = 0.007).

The population had an average age of 51 ± 13 years, and was 30% female. AF subjects had lower strain values vs. controls (17 ± 11 vs. 27 ± 10%), greater LA volumes (60 ± 18 vs. 35 ± 10) and lower LA EF (34 ± 18% vs. 52 ± 10%) (all p < 0.05). Average strain correlated well with decreasing LA volume and increasing LA EF (both R= ± 0.57, p=≤0.001), but correlated modestly with LGE burden (R=-0.36, p = 0.049) (Figure 1). In atrial regions of LGE enhancement, strain was lower, compared to regions without LGE (14 ± 11% vs. 20 ± 12%, p = 0.007), and a visual correspondence was clearly noted (Figure 2).

## Conclusions

In a cohort of mainly paroxysmal AF patients, global atrial strain was lower than in controls. The strains correlated strongly with LA EF and volumes, but modestly with LGE burden. However, on a regional basis, there was a correspondence between LGE enhancement and lower strain.
